# Centipede Venom Peptides Acting on Ion Channels

**DOI:** 10.3390/toxins12040230

**Published:** 2020-04-05

**Authors:** YanYan Chu, PeiJu Qiu, RiLei Yu

**Affiliations:** 1 School of Medicine and Pharmacy, Ocean University of China, 5 Yushan Road, Qingdao 266003, China; grfqiupeiju@hotmail.com; 2 Laboratory for Marine Drugs and Bioproducts, Qingdao National Laboratory for Marine Science and Technology, Qingdao 266003, China; 3 Innovation Center for Marine Drug Screening & Evaluation, Qingdao National Laboratory for Marine Science and Technology, Qingdao 266003, China; 4 Marine Biomedical Research Institute of Qingdao, Qingdao 266071, China

**Keywords:** animal toxin, ion channel, centipede venom, drug discovery, peptide drug

## Abstract

Centipedes are among the oldest venomous arthropods that use their venom to subdue the prey. The major components of centipede venom are a variety of low-molecular-weight peptide toxins that have evolved to target voltage-gated ion channels to interfere with the central system of prey and produce pain or paralysis for efficient hunting. Peptide toxins usually contain several intramolecular disulfide bonds, which confer chemical, thermal and biological stability. In addition, centipede peptides generally have novel structures and high potency and specificity and therefore hold great promise both as diagnostic tools and in the treatment of human disease. Here, we review the centipede peptide toxins with reported effects on ion channels, including Nav, Kv, Cav and the nonselective cation channel polymodal transient receptor potential vanilloid 1 (TRPV1).

## 1. Introduction

**Figure 1:**
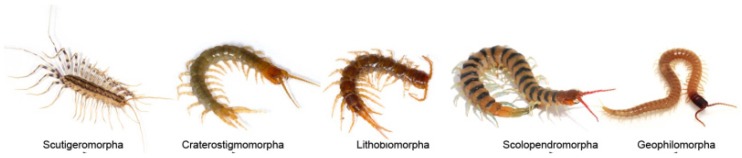
Pictures of five representative centipedes. All pictures are from the internet [7].

Centipedes, class Chilopoda, emerged approximately 440 million years ago [1] and are among the most ancient carnivorous terrestrial arthropods in soil ecosystems [2,3]. There are approximately 3300 species of centipede within five extant orders including Scutigeromorpha, Lithobiomorpha, Craterostigmomorpha, Scolopendromorpha and Geophilomorpha, which are distributed worldwide on all continents except Antarctica (Figure 1) [1,3,4]. The species in genus Scolopendra within Scolopendromorpha represents the best known centipedes because they are frequently involved in human accidents [5,6].

Centipedes prey mainly upon other arthropods by subduing them with venom injected via the forcipules, which stem from the first pair of legs [1]. The venom is also used to protect themselves from other predators or microorganisms. Centipede venom, containing large amounts of biogenic amines, serotonin, polysaccharides, lipids, peptide toxins and proteins [8], is a highly complex and functionally diverse mixture. For example, a glycosphingolipid from *Parafontaria laminata armigera* was reported to exert anticancer activity against melanoma cells by suppressing the focal adhesion kinase (FAK)-Akt pathway and the extracellular signal regulated kinase (Erk) 1/2 pathways [9].

For centuries, the centipede has been used in traditional medicines [10,11]. It was used to treat stroke-induced hemiplegia, epilepsy, apoplexy, whooping cough, tetanus, burns, tuberculosis, pain, arthritis, inflammation, tumors, and myocutaneous disease [11,12,13]. Thus, centipede venom is an important arsenal of new bioactive components that could be exploited for therapeutic use and drug development. However, unlike other venomous animals, such as snakes or spiders, centipedes are considered to be a neglected group, and little is known about their venom and their mechanism of action.

Centipede bites produce extremely sharp pain in humans [1,5]. Human victims bitten by centipedes usually experience intense burning pain, redness, swelling, chills, fever and weakness [8,14]. Large centipedes occasionally produce superficial necrosis, myocardial ischemia and infarction, hematuria, hemoglobinuria, rhabdomyolysis, hemorrhage, pruritus, eosinophilic cellulitis, and anaphylaxis, sometimes in conjunction with organ failure and acute coronary ischemia [15,16,17,18,19]. The numerous symptoms further indicate that centipede venom contains highly complex mixtures of diverse peptide toxins.

## 2. Centipede Toxins as an Abundant Source of Drug Leads

Peptide toxins have attracted increasing attention due to their excellent specificity for particular molecular targets and their extreme biomedical and pharmacological activities [20]. Peptide toxins generally have unusual structures and novel pharmacological properties and provide a rich source for the discovery of lead molecules, novel drugs, or new tools for ion channel manipulation. Currently, there are six FDA-approved drugs derived from venoms for the treatment of diabetes, hypertension and chronic pain [20,21,22,23,24,25,26,27]. Many additional venom-derived molecules are under clinical and preclinical development [28,29,30,31,32,33,34,35,36,37,38].

Centipede venom contains an abundance of peptide toxins. With advances in transcriptomics and proteomics, the composition of centipede venom was confirmed through continuing effort [8,10,39,40]. Liu et al. profiled the venom proteome and gland transcriptome from *Scolopendra subspinipes dehaani* and characterized 40 peptides [39]. Rong et al. also focused on the diversity of centipede venom and obtained 79 unique peptide toxins from the venom of *Scolopendra subspinipes mutilans* [10]. Recently, Zhao et al. identified 1204 unique proteins in the torso tissue and 165 unique proteins in the venom gland peptides of *Scolopendra subspinipes mutilans* using proteotranscriptomics [8].

Centipede peptides generally bear no resemblance to any characterized peptide family, highlighting the novelty of centipede venoms [7]. Phylogenetically, centipede peptides are divided into 31 families, SCUTX_1-3_ and SLPTX_1-28_. Among them, 24 families, including SCUTX_1-2_, SLPTX_1-20_, SLPTX_26_ and SLPTX_28_, comprise cysteine-rich peptides with one to eight putative disulfide bonds (Table 1) [1,10,41]. The multiple disulfide bonds confer high chemical, thermal and biological stability on the peptides, enabling researchers to exploit various desirable functions.

Centipede peptide toxins exhibit a variety of biomedical and pharmacological activities—currently, approximately 50 components of centipede venom have been reported with properties including ion channel activity, antimicrobial activities, platelet-aggregating activity, anticoagulant activity, phospholipase A2 activity, and trypsin-inhibiting activity [7,10,39,40,42,43,47,49,52,53,54]. Most centipede peptide toxins are neurotoxins that act on ion channels, including voltage-gated sodium channels (VGSCs), voltage-gated potassium channels, voltage-gated calcium channels, and polymodal transient receptor potential vanilloid 1 (TRPV1) [39,42,43,49]. 

### 2.1. Voltage-Gated Sodium Channel (Nav) Blocker

Navs are essential for the rapid upstroke of action potentials and the propagation of electrical signals in nerves and muscles. They are closely associated with a variety of diseases, including epilepsy, cardiac arrhythmias, and neuropathic pain [55,56], and therefore have been regarded as appealing therapeutic targets for the development of anticonvulsant, antiarrhythmic, and local anesthetic drugs [57,58].

**Figure 2:**
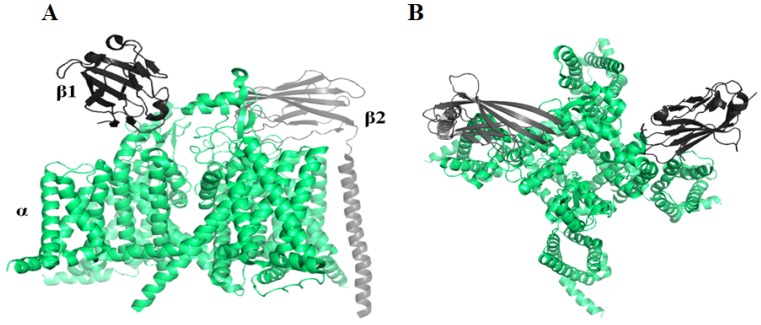
(**A**) The side view of Nav1.7 (PDB ID: 6J8I [67]) containing one α subunit and two β subunits. (**B**) The Nav1.7 structure from a top-down view of the tetrameric channel.

Navs are composed of one α subunit and one or more β subunits (Figure 2). In mammals, Nav channels have nine known alpha members, Nav1.1-Nav1.9, which are selectively expressed in dorsal root ganglia (DRG) neurons [59]. Pharmacologically, Navs may be classified by their sensitivity to the neurotoxin tetrodotoxin (TTX). Nav1.5, Nav1.8 and Nav1.9 are TTX-resistant (TTX-R), while other subtypes are TTX-sensitive (TTX-S) [60]. Nav1.7, Nav1.8, and Nav1.9, predominantly expressed in peripheral neurons, are important targets for chronic pain therapy [61,62,63,64].

All α subunits share high sequence conservation and nearly identical structure topology. Thus, the design of isoform-selective Nav modulators is challenging [65]. Two centipede peptides, μ-SLPTX_3_-Ssm2a and μ-SLPTX_3_-Ssm3a, were identified as specific Nav blockers [42,66].

#### 2.1.1. μ-SLPTX_3_-Ssm2a

**Figure 3:**
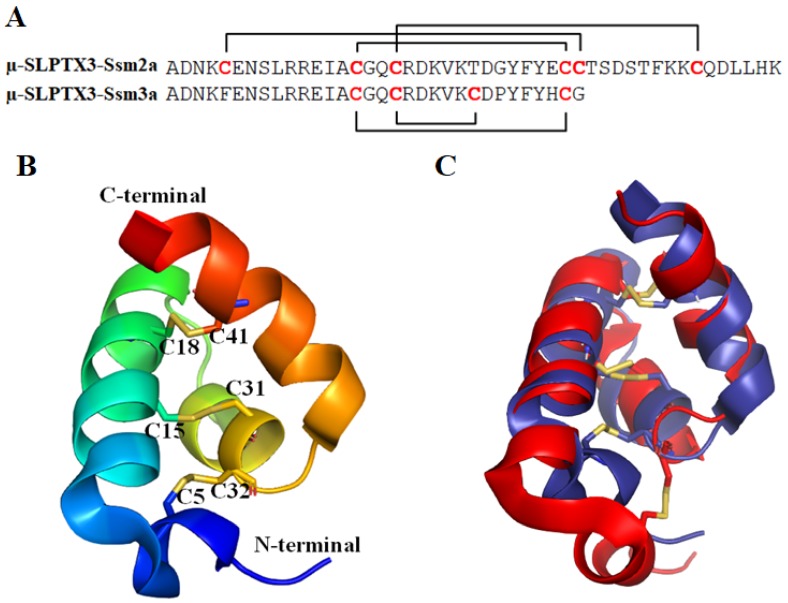
(**A**) The sequences of µ-SLPTX3-Ssm2a and µ-SLPTX3-Ssm3a. (**B**) The structure of µ-SLPTX3-Ssm2a (PDB ID: 2MUN [66]). The cysteine pairs forming disulfide bonds are labeled and shown in stick representation. (**C**) Overlay of µ-SLPTX3-Ssm2a (dark blue) and Ta1a (red, PDB ID: 2KSL [66]). The disulfide bonds are shown in stick representation.

µ-SLPTX_3_-Ssm2a (also named µ-SLPTX-Ssm6a) has a mass of 5318.4 Da. It is a 46-amino-acid peptide whose structure was determined using heteronuclear NMR (Figure 3), revealing an exclusively α-helical structure comprised of four helices. It is structurally homologous to the spider toxin Ta1a [66]. The helices are crossbraced by three intramolecular disulfide bonds formed between C5–C32, C15–C31 and C18–C41 (PDB ID: 2MUN [66], Figure 3). The unique helical structure constitutes a new structural class of venom toxins, referred to as helical arthropod-neuropeptide-derived (HAND) toxins [66].

Yang et al. reported that the peptide completely inhibited the TTX-S currents at 1 µM but had no effect on the TTX-R currents at 10 µM. Further research found that µ-SLPTX_3_-Ssm2a potently and selectively inhibits Nav1.7 with an IC_50_ of 25.4 nM. The IC_50_ values over key off-target Nav subtypes were 4.1 µM for Nav1.1, 813 nM for Nav1.2, and 15.2 µM for Nav1.6 [42]. The peptide toxin had no effect on Nav1.3, Nav1.4, Nav1.5, Nav1.8 and hERG (Kv11.1) [42]. Thus, µ-SLPTX_3_-Ssm2a has more than 30-fold selectivity over Nav1.2 and more than 150-fold selectivity over Nav1.1, Nav1.6 and other Nav subtypes. 

In vivo, µ-SLPTX_3_-Ssm2a was a more effective analgesic than morphine in a rodent pain model of chemical-induced pain and was equipotent with morphine in rodent models of thermal- and acid-induced pain. Moreover, the peptide toxin was highly stable in human plasma and had no evident adverse effects on blood pressure, heart rate, or motor function at a dose of 1 µmol/kg. All the above data indicated that µ-SLPTX_3_-Ssm2a is a promising candidate for the development of novel analgesics specifically targeting Nav1.7 [42]. However, two teams found independently that the synthesized µ-SLPTX_3_-Ssm2a was inactive against Nav1.7 at concentrations up to 1 µM [68,69]. This suggested that there should be other compounds in the native fraction contributing to the bioactivity. 

#### 2.1.2. μ-SLPTX_3_-Ssm3a

μ-SLPTX_3_-Ssm3a [42] is a peptide toxin of 32 amino acids with a molecular mass of 3762.5 Da (Figure 3). It was first identified in *Scolopendra subspinipes mutilans* by Yang et al. and named μ-SLPTX-Ssm1a [42]. It contains four cysteine residues forming two disulfide bonds. μ-SLPTX_3_-Ssm3a was observed to specifically inhibit the TTX-S Na_v_ channel current in rat DRGs with an IC_50_ of ~9 nM [11,43]. Ten micromolar μ-SLPTX_3_-Ssm3a inhibited the TTX-S Nav current amplitude by almost 100% but had no effect on TTX-R Nav currents [42]. In vivo, μ-SLPTX_3_-Ssm3a had potent insecticidal activities against adult blowflies, mealworms and cockroaches. The LD_50_ values ranged from 67 pmol/g (0.25 μg/g) in adult blowflies to 6300 pmol/g (23.7 μg/g) in cockroaches [42]. The excellent biomedical activity and strong specificity make μ-SLPTX_3_-Ssm3a a potential lead for therapeutic application or pesticide development.

### 2.2. Voltage-Gated Potassium Channel (Kv) Inhibitor

**Figure 4:**
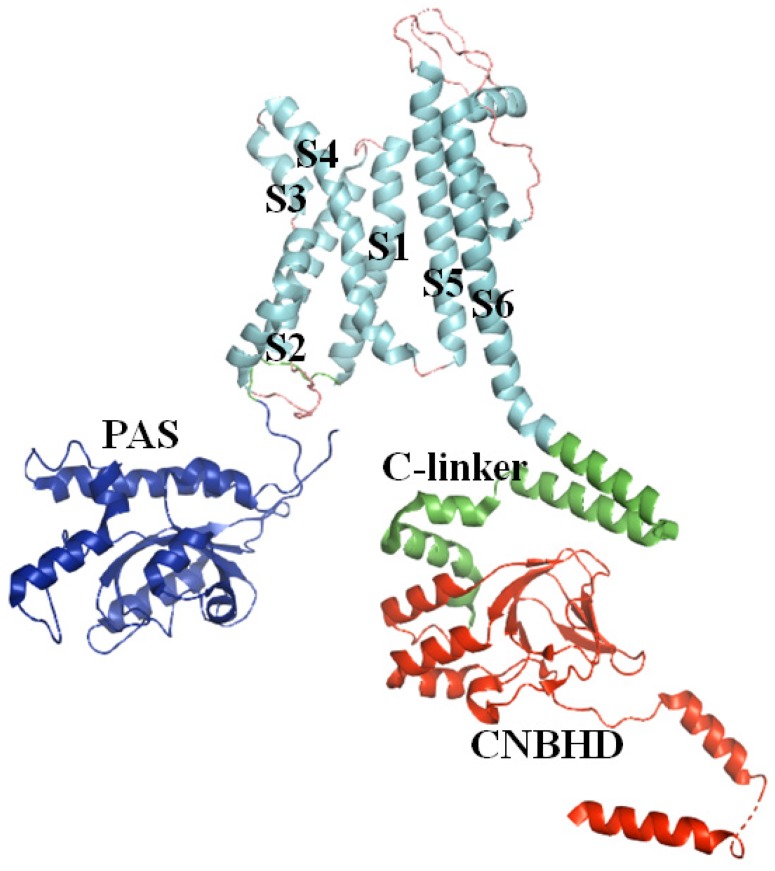
The structure of rat Kv10.1 (PDB ID: 5K7L [71]). The key information is labeled.

Kv channels are transmembrane channels specific for potassium. There are 12 members of the Kv channel family, Kv1–Kv12. Most are homogeneous tetramers, and each subunit is comprised of six transmembrane segments S1–S6 (Figure 4). Segments S1–S4 form the voltage sensor (VS) that activates upon membrane depolarization. The movement of VS is coupled to the K^+^ selective pore (S5–S6) by a helical S4–S5 linker [70]. The Eag family, which includes Kv10–Kv12, contains three intracellular domains, an N-terminal Per-ARNT-Sim (PAS) domain, a C-terminal C-linker domain, and a C-terminal cyclic nucleotide binding homology domain (CNBHD). The PAS domain is also important in gating.

Kvs play key roles in a variety of cellular processes, including the functioning of excitable cells, regulation of apoptosis, cell growth and differentiation, release of neurotransmitters and hormones, and maintenance of cardiac activity [72]. Mutations in Kv channel genes are related to hereditary disorders, cardiac rhythm disorders, sclerosis and pain. Therefore, Kv channels are regarded as prospective drug targets. Several centipede toxins have been identified as Kv inhibitors, including κ-SLPTX3-Ssm1a, κ-SLPTX-Ssm2a, κ-SLPTX_11_-Ssm3a, κ-SLPTX15-Ssd2a, SsmTx-1, SsTx, and SSD609.

#### 2.2.1. κ-SLPTX_3_-Ssm1a

**Figure 5:**
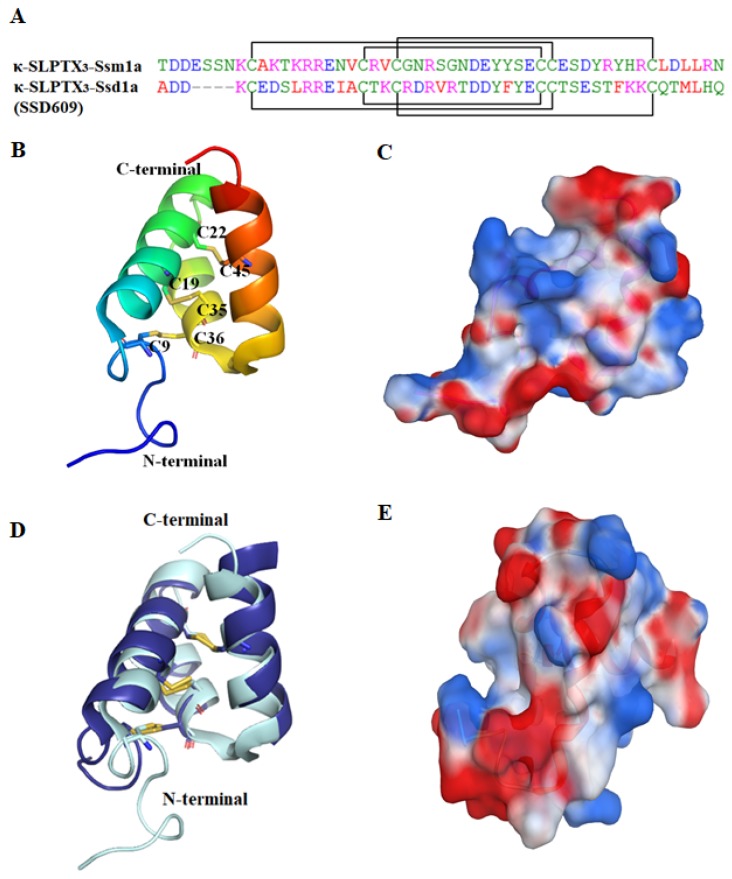
The sequence and structure of κ-SLPTX_3_-Ssm1a (PDB ID: 2M35) and SSD609 (PDB ID: 2MVT [44]). (**A**) The sequences of κ-SLPTX_3_-Ssm1a and SSD609. The cysteine pairs forming disulfide bonds are connected with square brackets. (**B**) The cartoon structure of κ-SLPTX_3_-Ssm1a (PDB ID: 2M35). The four cysteine residues are shown in stick representation. (**C**) The electrostatic surface of κ-SLPTX_3_-Ssm1a. (**D**) Overlay of κ-SLPTX_3_-Ssm1a (light cyan) and SSD609 (dark blue). (**E**) The electrostatic surface of SSD609. The positive and negative electrostatic potential was shown in blue and red, respectively.

κ-SLPTX_3_-Ssm1a [1] (also named κ-SLPTX-Ssm1a) is a polypeptide toxin with a mass of 6050.2 Da. It contains 51 amino acids, and the sequence is homologous with that of κ-SLPTX-Ssm1b-1e [43]. The NMR structure showed that the peptide is structurally compact with a flexible N-terminal, three α-helices and two loops (PDB ID: 2M35, Figure 5B). The side chains of the polar residues are oriented toward the lateral side of the peptide, giving the structure strong polarity (Figure 5C). There are three disulfide bonds between C9–C36, C19–C35 and C22–C45. The disulfide connectivity pattern (1/5, 2/4, 3/6, where 1/5 refers to the 1st cysteine connected with the 5^th^ cysteine, and so on) is the same as that of another centipede toxin, SSD609.

κ-SLPTX_3_-Ssm1a is a Kv inhibitor. It inhibits the Kv current in DRG neurons with an IC_50_ of approximately 44.2 nM [43]. In vivo, insects injected with this toxin showed signs of neurotoxicity, including twitching, paralysis, and body contraction. Thus, κ-SLPTX_3_-Ssm1a exhibited potent insecticidal activity against adult blowflies, with an LD_50_ of 12.5 pmol/g (0.076 μg/g) [43].

#### 2.2.2. SSD609

SSD609 (named κ-SLPTX_3_- Ssd1a) is a polypeptide toxin from *Scolopendra subspinipes dehaani* (SSD). SSD609, with a molecular weight of 5624.5 Da [39], consists of 47 amino acids. Six cysteines form three disulfide bonds between C5–C32, C15–C31 and C18–C41.

The structure of SSD609 was characterized using solution nuclear magnetic resonance (PDB ID: 2MVT [44]). As mentioned above, it is a distinct three-helix peptide with a special disulfide connectivity pattern (Figure 5D). The peptide also shows strong polarity, which probably contributes to its solubility. Although SSD609 and κ-SLPTX_3_-Ssm1a are similar in sequence, their molecular shapes are obviously different (Figure 5C,E). The special architecture provides a distinctive action mechanism.

SSD609 is the first toxin peptide known to target KCNE1, which is a single-span transmembrane auxiliary protein that regulates KCNQ1 (Kv7.1) by slowing its activation/deactivation kinetics and increasing KCNQ1 current amplitude [73,74,75]. The KCNQ1/KCNE1 complex is an essential component in cardiac myocytes that regulates heart rhythms and underlies the cardiac slow delayed rectifier potassium current (*I*_Ks_). SSD609 reversibly inhibited the channel conductance of I*_ks_* with an IC_50_ of 652.7 nM and had no obvious inhibitory effect on KCNQ1 alone, KCNQ1/KCNE2 or KCNQ/KCNE4 channels expressed in Chinese hamster ovary (CHO) cells [39]. Therefore, Sun et al. proposed that SSD609 specifically interacts with KCNE1. Structural and functional analysis indicated that E19 of KCNE1 was the key residue participating in the direct interaction with SSD609 [44]. However, Ombati et al. [50] questioned the proposal and suggested that SSD609 modulates only the current to the α subunit of the KCNQ family because KCNE1 is not a key component in channel voltage activation [76].

#### 2.2.3. SsTx

Ssm Spooky Toxin (SsTx) (also named μ-SLPTX_15_-Ssm1a), with a mass of 6017.5 Da, is a 53-amino-acid peptide identified in golden head centipedes (*Scolopendra subspinipes mutilans*). Lethal toxicity was observed, indicating key roles in paralyzing prey. The toxicity could be neutralized by retigabine, a Kv7 opener [45].

**Figure 6:**
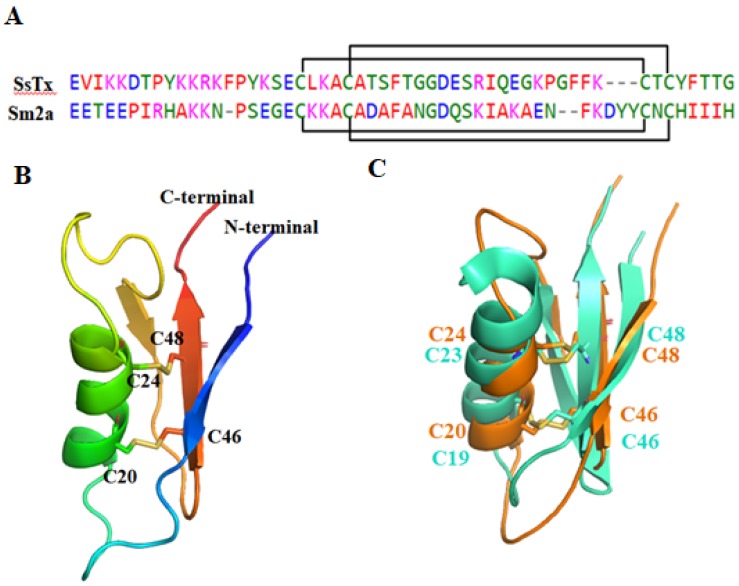
(**A**) The sequences of SsTx and U-SLPTX_15_-Sm2a (Sm2a). The cysteine pairs forming disulfide bonds are connected with square brackets. (**B**) The structure of SsTx. (**C**) Structural superposition of SsTx (orange cartoon) and U-SLPTX_15_-Sm2a (green cartoon).

The structure of SsTx (PDB ID: 5X0S) was recently elucidated by Luo et al. (Figure 6) [45]. SsTx has two disulfide bonds between C20-C46 and C24-C53 [46]. It adopts a novel structural arrangement called 2ds-CSα/β, which consists of an α-helix connected to a β-sheet by two disulfide bonds (CSα/β) [77]. The 3D structure of SsTx is similar to that of U-SLPTX_15_-Sm2a, which is a centipede peptide without antimicrobial activity, Kv activity, Nav activity or Cav activity [45].

SsTx exhibited potent inhibitory activity on K_V_7 and it did not inhibit channels TRPV1 and TRPV2, Kv2.1 and Kv4.1, hERG, TTX-S and TTX-R Nav or Cav in DRG neurons [45]. SsTx inhibited Kv7 with IC_50_ values of 2.5 µM for Kv7.4, 2.8 µM for Kv7.1, 2.7 µM for Kv7.2 and 2.7 µM for Kv7.5 [45]. Recently, Du et al. reported that SsTx also inhibited Kv1.3 channels in a voltage-dependent manner, with an IC_50_ value of 5.26 µM [46].

Structural and functional assays of the interaction between SsTx and Kv7.4 revealed that all of the basic residues on SsTx contributed to the inhibitory effect on Kv7.4. The inhibitory effect of R12A and K13A mutants on Kv7.4 was significantly reduced by approximately 20-fold. Therefore, R12 and K13 are two key residues responsible for the interaction with the Kv7.4 channel. The side chain of K13 anchors the peptide to the outer pore region of Kv7.4, and R12 extends into the selectivity filter. Further assays identified Kv7.4 residues D266 in the turret and D288 in the P-loop region, which are conserved among all subtypes of Kv7, are crucial for SsTx binding [46]. A peptide toxin interaction study revealed that K13-D266 and R12-D288 are two interacting residue pairs critical for the centipede toxin’s functional activity on Kv7.4 [46,50]. In contrast to the interaction with Kv7, K13 and K11 have been found to contribute to SsTx binding to Kv1.3. Alanine substitution of either of the two residues increased the IC_50_ values by more than 100-fold [46]. The R12A mutant selectively inhibited Kv1.3 channels. Kv1.3 is expressed abundantly in immune cells and is a target for curing autoimmune diseases. Thus, SsTx^R12A^ is a potential drug for curing autoimmune diseases [45,46]. 

In vivo, SsTx exhibits abundant pharmacological activities. It affects the cardiovascular system and exerts vasoconstrictive activity, resulting in acute hypertension and sometimes coronary-induced vasospasms, ultimately leading to heart failure when injected intravenously in mice and Macaca monkeys [45]. SsTx can induce seizures when injected into the hippocampus of mice [45]. It also causes disorders of the nervous and respiratory systems [45,46].

#### 2.2.4. κ-SLPTX_7_-Ssm2a

**Figure 7:**
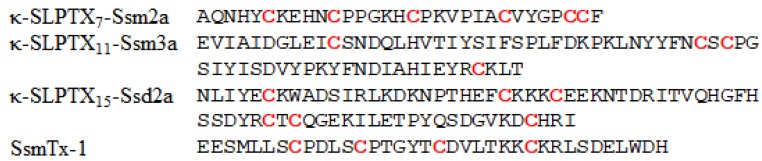
The sequences of other known centipede peptides acting on the Kv channel.

κ-SLPTX7-Ssm2a (also named κ-SLPTX_7_-Ssm2a) is a 31-amino-acid peptide with a molecular mass of 3465.8 Da (Figure 7), which is much smaller than κ-SLPTX_3_-Ssm1a. It contains six cysteines forming three disulfide bonds. The structure has not been determined. Similar to κ-SLPTX_3_-Ssm1a, κ-SLPTX_7_-Ssm2a inhibits K_V_ currents in DRG neurons with an IC_50_ of ~570 nM. In vivo, κ-SLPTX-Ssm2a has insecticidal activities, and the LD_50_ against adult blowflies is 5 pmol/g (0.017 μg/g) [43].

#### 2.2.5. κ-SLPTX_11_-Ssm3a

κ-SLPTX_11_-Ssm3a is also a K_V_ channel inhibitor. It is a 68-amino-acid peptide with a molecular mass of 7989.07 Da (Figure 7), and four cysteine residues that form two disulfide bonds. Phylogenetic analysis revealed that κ-SLPTX_11_-Ssm3a is a truncated form of a family dominated by cysteine-rich proteins with molecular weights of ~20 kDa [1,7]. Undheim et al. suggested that the peptide belongs to the same family as three high-molecular-weight Kv inhibitors containing up to 16 cysteine residues [1]. κ-SLPTX_11_-Ssm3a inhibits Kv current amplitude by 25% at a concentration of 200 nM, and it does not fully inhibit peak Kv currents even at concentrations up to 5 µM [43]. However, this toxin showed more potent inhibitory activity against slowly activating rectifier K^+^ currents. Thus, κ-SLPTX_11_-Ssm3a could be complementary to the activity of other toxins that inhibit peak current. In vivo, κ-SLPTX_11_-Ssm3a showed potent insecticidal activities against adult blowflies, with an LD_50_ of 5 pmol/g (0.040 μg/g) [43].

#### 2.2.6. κ-SLPTX_15_-Ssd2a

κ-SLPTX_15_-Ssd2a with a mass of 8556.2 Da was identified in *Scolopendra subspinipes dehaani*. It is composed of 72 amino acids, including six cysteine residues (Figure 7). κ-SLPTX_15_-Ssd2a irreversibly blocks K_V_ currents with an estimated IC_50_ of approximately 10 nM in DRG neurons [39].

#### 2.2.7. SsmTx-1

SsmTx-1, a 36-amino-acid peptide with a mass of 4114.068 Da (Figure 7), was first isolated from the venom of *Scolopendra subspinipes mutilans* [47] and contains two disulfide bonds between C8–C19 and C13-C26 [48]. SsmTx-1 can potently and selectively block Kv channels in DRGs instead of Nav channels, with an IC_50_ of 200 nM. Among nine K^+^ subtypes expressed in human embryonic kidney 293 cells, SsmTx-I selectively blocked the K_v_2.1 current with an IC_50_ value of 41.7 nM [47]. In vivo, SsmTx-I showed potential analgesic activities in formalin-induced paw licking, thermal pain, and acetic acid-induced abdominal writhing mouse models [48].

### 2.3. Voltage-Gated Calcium Channel (Cav) Modulator

Tuned calcium entry through Cavs is a key requirement for many cellular functions, such as the plateau of the cardiac action potential, contraction of muscle cells, generation of pacemaker potentials, release of hormones and neurotransmitters, sensory functions, and gene expression [78,79,80]. There are ten members of the Cav family in mammals—high-voltage-activated channels Cav1.1–1.4 and Cav2.1–2.3 and low-voltage-activated channels Cav3.1–3.3 [78]. Different members play distinct roles in cellular signal transduction.

**Figure 8:**
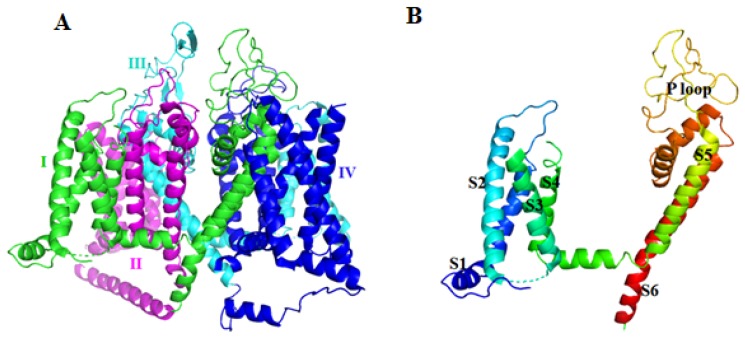
The structure of the α1 subunit of rabbit Cav1.1 (PDB ID: 6BYO [83]). (**A**) The cartoon is colored by domain. (**B**) Structure of domain I.

The pore-forming transmembrane α1 subunit of Cav is organized into four homologous domains (I–IV), each comprised of six transmembrane α helices (S1–S6) and the pore-forming P-loop between S5 and S6 (Figure 8) [81,82]. Structural and functional analysis indicated that S4 segments form a key part of the VS module.

Cav channels are closely related to several diseases. Mutations in the Cav gene cause hypokalemic periodic paralysis, migraine headache, psychiatric disorder, cardiac arrhythmia, autism, and developmental abnormalities [79,84,85,86]. To date, two centipede peptides, ω-SLPTX_5_-Ssm1a and ω-SLPTX_13_-Ssm2a, have been identified as Cav channel modulators.

#### 2.3.1. ω-SLPTX_5_-Ssm1a

**Figure 9:**
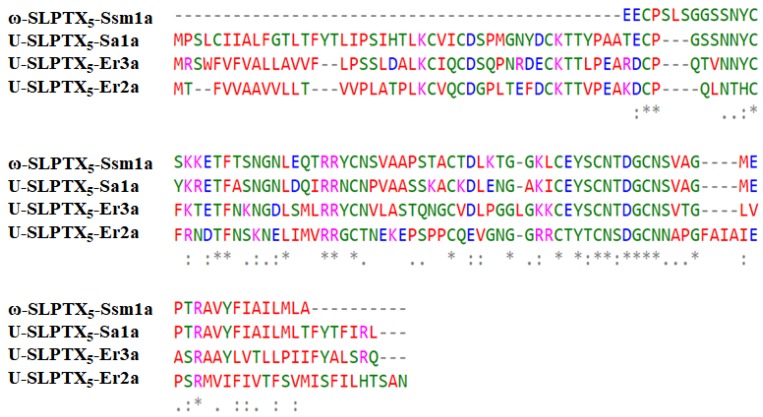
Sequence alignment of ω-SLPTX_5_-Ssm1a with three homologous peptides.

ω-SLPTX_5_-Ssm1a has a molecular mass of 8810.4 Da and was identified in *Scolopendrinae* [7,43]. It comprises 86 amino acid residues (Figure 9). This peptide is special because it contains an odd number of cysteine residues. Six of the seven cysteines form three intramolecular disulfide bonds. ω-SLPTX_5_-Ssm1a shares sequence homology with three centipede toxins, U-SLPTX5-Sa1a, U-SLPTX5-Er3a and U-SLPTX5-Er2a, with sequence identities of 82%, 47.5% and 43.2%, respectively. ω-SLPTX5-Ssm1a acts as an activator of Cav channels in DRG neurons. It was reported that 1 μM ω-SLPTX_5_-Ssm1a increased Ca_V_ currents in DRG neurons by 70%, whereas 10 μM ω-SLPTX_5_-Ssm1a increased Cav currents by 120% [43].

#### 2.3.2. ω-SLPTX_13_-Ssm2a

**Figure 10:**
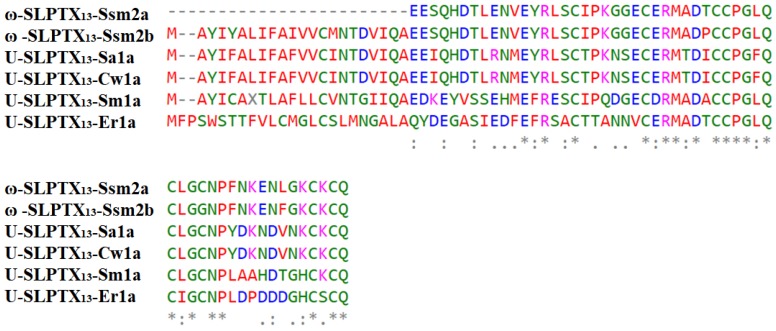
Sequence alignment of ω-SLPTX_13_-Ssm2a with its homologous peptides.

ω-SLPTX_13_-Ssm2a has a mass of 6014.2 Da and contains 54 residues and eight cysteines that form four disulfide bonds (Figure 10). The reported sequence of ω-SLPTX_13_-Ssm2a is similar to several spider lycotoxins, with 41% sequence identity [43]. Sequence analysis showed similarity to several other centipede peptides, ω-SLPTX_13_-Ssm2b, U-SLPTX_13_-Sa1a, U-SLPTX_13_-Cw1a, U-SLPTX_13_-Sm1a and U-SLPTX_13_-Er1a, with sequence identities of 96.1%, 76.3%, 76.3%, 57.9% and 44.9%, respectively. ω-SLPTX-Ssm2a inhibits Cav channel currents in DRG neurons with an IC_50_ of approximately 1590 nM [43].

### 2.4. TRPV1 Activator

The capsaicin receptor TRPV1 is a nonselective cation channel located in the plasma membrane of nociceptive DRG neurons. It is a polymodal nociceptor that responds to heat with exquisite sensitivity and is involved in detecting the surrounding environment to maintain stable body temperature in mammals and in heat pain transduction [49,87,88]. 

**Figure 11:**
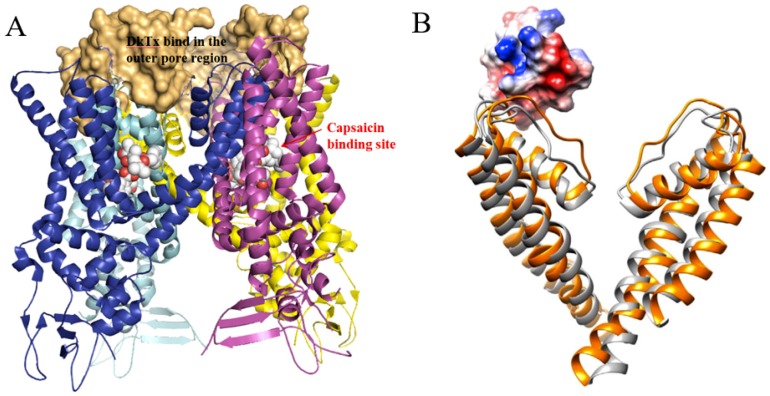
The structure of polymodal transient receptor potential vanilloid 1 (TRPV1). (**A**) The crystal structure of TRPV1 tetramer binding with the spider peptide toxin DkTx. The cartoon is colored by TRPV1 subunits. The molecular surface in orange represents DkTx, which is bound to the outer pore region. (**B**) A docking model of TRPV1 monomer (orange ribbon) bound with centipede toxin RhTx (surface) by Yang et al. [49]. TRPV1 in the closed state (grey ribbon) was overlaid to the model.

TRPV1 is a homotetrameric structure (Figure 11). Each of the four subunits is composed of six transmembrane segments, S1–S6, with a pore-forming loop between S5 and S6 [51,89]. There are two major TRPV1 binding sites responsible for TRPV1’s capacity to respond to a multitude of agonists, antagonists, and channel blockers. One is the capsaicin binding site located in S3–S4, and the other is the outer pore region, which is essential for binding peptide toxins, such as DkTx [51] and RhTx [49].

TRPV1 is closely related to various types of pain, including inflammatory pain, neuropathic pain, and cancer pain [90,91,92,93]. RhTx is the only centipede toxin that has been reported to activate the TRPV1 channel.

#### RhTx

RhTx (named τ-SLPTX_4_-Sm1a) is a 27-amino-acid peptide toxin with a molecular mass of 2967.3 Da and was identified in the venom of the Chinese red-headed centipede *Scolopendra subspinipes mutilans*. This peptide can produce excruciating pain by potently activating the nociceptor TRPV1. RhTx is a selective TRPV1 activator with an EC_50_ of 521.5 nM and does not affect other TRPV channels [28,49,50,51].

**Figure 12:**
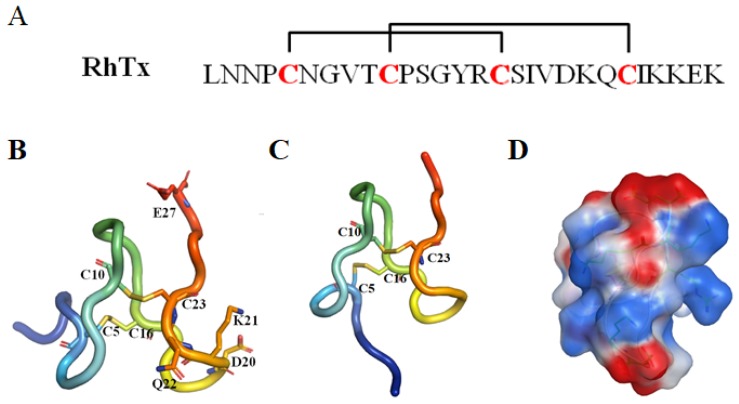
(**A**) The sequence of RhTx. The cysteine pairs forming disulfide bonds are connected with square brackets. (**B**) and (**C**) Two representative conformations of RhTx (PDB ID: 2MVA). The key residues are labeled and shown in stick representation. (**D**) The electrostatic surface of RhTx. The positive and negative electrostatic potential was shown in blue and red, respectively.

NMR spectroscopy analysis indicated that RhTx contains four cysteines forming two intramolecular disulfide bonds, C5–C16 and C10–C23, with a pattern typical of the SLPTX4 family [28]. RhTx folds into a compact structure with a flexible N-terminal tail (PDB ID: 2MVA, Figure 12). Most of the charged residues located in the C-terminus and the charged side chain are exposed, making RhTx a polarized molecule.

The structure and functional investigation indicated that the charged C-terminus can interact directly with the charge-rich outer pore of TRPV1. TRPV1 residues D602 in the turret, Y632 and T634 in the pore helix and L461 are critical for RhTx-induced channel activation. Moreover, the outer pore is a known hot spot mediating the action of many chemical activators, such as H^+^ [94,95], divalent cations [96,97] and spider toxin DkTx [98]. Systematic functional examination indicated that RhTx strongly promotes the heat activation process by decreasing the activation threshold temperature. RhTx exhibits rapid binding kinetics and high binding affinity for TRPV1, comparable to that of the 75-amino-acid peptide DkTx [98]. RhTx activates TRPV1 through an allosteric mechanism and promotes TRPV1 opening by binding preferentially to the activated state. RhTx does not bind to the closed state of TRPV1 since it is ineffective when the channel is held closed by cooling. Alanine substitution at each of the 23 noncysteine positions showed that four mutants (D20A, K21A, Q22A and E27A) decreased the binding affinity to TRPV1, while R15A enhanced the apparent binding affinity [49]. This study makes RhTx a potential candidate for development as a new drug to treat pain.

## 3. Conclusions

Centipede venom represents an important arsenal of new bioactive components. Most peptide toxins act on voltage-gated ion channels. Centipede peptides can interfere with Nav, Kv, Cav and TRPV1 channels, which is consistent with the numerous symptoms of centipede bites and the abundant roles of centipedes in traditional medicine. Four out of twelve peptide toxins, µ-SLPTX_3_-Ssm2a, SsmTx-1, SsTX and RhTx, exhibit excellent target specificity. Most peptide toxins can block ion channel currents. However, two centipede peptides, ω-SLPTX_5_-Ssm1a and RhTx, are instead activators, making them essential pharmacological tools. The structures of centipede peptide toxins exhibit novel structural arrangements (µ-SLPTX_3_-Ssm2a in HAND and SsTx in 2ds-CSα/β), characteristic disulfide connectivity patterns (κ-SLPTX_3_-Ssm1a and SSD609) and in one case an odd number of cysteine residues (ω-SLPTX_5_-Ssm1a). All of these features indicate that centipede peptide toxins hold promise as diagnostic tools and therapeutic candidates.

The current research on centipede toxins is still far from sufficient. Only 12 toxins with known sequences were tested for ion channel activities. Many more neurotoxins have been identified by transcriptomics and proteomics and need to be further elucidated. With rapid technological development, more bioactive peptides are expected to be identified soon.

## Figures and Tables

**Table 1:** Features and functionally described components from centipedes.

Peptide Toxin (Other Name)	Number of Residues	Disulfide/Cysteine Numbers (Cysteine Pairs)	Bioactivity
μ-SLPTX_3_-Ssm2a (µ-SLPTX-Ssm6a)	46	3/6 (C5–C32, C15–C31, C18–C41)	Nav1.7, IC_50_ = 25.4 nM; Nav1.1, IC_50_ = 4.1 µM; Nav1.2, IC_50_ = 813 nM; Nav1.6, IC_50_ = 15.2 µM. No effect on Nav1.3, Nav1.4, Nav1.5, Nav1.8 and hERG [42].
μ-SLPTX_3_-Ssm3a (μ-SLPTX-Ssm1a)	32	2/4 (-)	Specifically inhibited TTX-S Nav channel current in rat DRGs, IC_50_ = ~9 nM [11,43]. No effect on TTX-R Nav.
κ-SLPTX_3_-Ssm1a (κ-SLPTX-Ssm1a)	51	3/6 (C9–C36, C19–C35, C22–C45)	Inhibited Kv current in DRG neurons, IC_50_ = ~44.2 nM [43].
SSD609 (κ-SLPTX_3_- Ssd1a)	47	3/6 (C5–C32, C15–C31, C18–C41)	Inhibited the channel conductance of I*ks*, IC_50_ = 652.7 nM [39,44].
SsTx (μ-SLPTX_15_-Ssm1a)	53	2/4 (C20–C46, C24–C53)	K_V_7.4, IC_50_ = 2.5 µM; Kv7.1, IC_50_ = 2.8 µM; Kv7.2, IC_50_ = 2.7 µM; Kv7.5, IC_50_ = 2.7 µM; Kv1.3, IC_50_ = 5.26 µM; no inhibition of TRPV1, TRPV2, Kv2.1, Kv4.1, hERG, Nav or Cav in DRG neurons [45,46].
κ-SLPTX_7_-Ssm2a (κ-SLPTX_7_-Ssm2a)	31	3/6 (-)	K_V,_ IC_50_ = ~570 nM [43].
κ-SLPTX_11_-Ssm3a	68	2/4 (-)	Inhibited Kv current amplitude by 25% at a concentration of 200 nM, did not fully inhibit peak Kv currents even at concentrations up to 5 µM [43].
κ-SLPTX_15_-Ssd2a	72	-/6 (-)	Irreversibly blocked K_V_ currents, IC_50_ = ~10 nM [39].
SsmTx-1	36	2/4 (C8–C19, C13–C26)	Kv, IC_50_ = 200 nM; Kv2.1, IC_50_ = 41.7 nM. No effect on Nav [47,48].
ω-SLPTX_5_-Ssm1a	86	3/7 (-)	Cav activator, 1 μM ω-SLPTX_5_-Ssm1a increased Ca_V_ current in DRG neurons by 70%; 10 μM toxin increased Cav current by 120% [7,43].
ω-SLPTX_13_-Ssm2a	54	4/8 (-)	Cav, IC_50_ = 1590 nM [43].
RhTx (τ-SLPTX_4_-Sm1a)	27	2/4 (C5–C16, C10–C23)	TRPV1 activator, EC_50_ = 521.5 nM. No effect on other TRPV channels [28,49,50,51].

Note: (-) means the disulfide connectivity pattern is unknown.
